# *Toxoplasma gondii* GRA8 induces ATP5A1–SIRT3-mediated mitochondrial metabolic resuscitation: a potential therapy for sepsis

**DOI:** 10.1038/emm.2017.308

**Published:** 2018-03-30

**Authors:** Ye-Ram Kim, Jae-Sung Kim, Jin-Seung Yun, Sojin Kim, Sun Young Kim, Kiseok Jang, Chul-Su Yang

**Affiliations:** 1 Department of Molecular and Life Science, Hanyang University, Ansan, South Korea; 2 Department of Bionano Technology, Hanyang University, Seoul, South Korea; 3 Department of Pathology, Hanyang University College of Medicine, Seoul, South Korea

## Abstract

The intracellular parasite *Toxoplasma gondii* has unique dense granule antigens (GRAs) that are crucial for host infection. Emerging evidence suggests that GRA8 of *T. gondii* is a promising serodiagnostic marker in toxoplasmosis. However, little is known about the intracellular regulatory mechanisms involved in GRA8-induced host responses. We found that GRA8 interacts with host proteins involved in mitochondria activation and might be useful as a therapeutic strategy for sepsis. Here, we show that protein kinase-Cα (PKCα)-mediated phosphorylation of *T. gondii* GRA8 (Thr220) is required for mitochondrial trafficking and regulates the interaction of C terminal of GRA8 with nucleotide binding domain of ATP5A1. Furthermore, GRA8 interacts with SIRT3 in mitochondria, facilitating ATP5A1 deacetylation (K506 and K531), adenosine triphosphate production and subsequent anti-septic activity *in vivo*. Taken together, these results demonstrate a new anti-sepsis therapeutic strategy using *T. gondii* GRA8-induced mitochondrial metabolic resuscitation. This strategy represents an urgently needed paradigm shift for therapeutic intervention.

## Introduction

The protozoan parasite *Toxoplasma gondii* is the infectious agent of one of the most common zoonoses worldwide and causes toxoplasmosis. It causes development of parasitophorous vacuoles after invading host cells, accompanied by the release of 11 excretory–secretory *T. gondii* dense granule proteins (GRAs).^1, 2^ GRAs have an N-terminal signal sequence, and GRAs 5–8 have an internal hydrophobic domain that presumably anchors these proteins in parasitophorous vacuoles.^1^ GRA8 is an acute phase-specific antigen that allows survival of the parasite within host cells, and it is a promising vaccine candidate for toxoplasmosis.^1, 3^ Studies using the yeast two-hybrid technique have shown interactions between GRA8 and various host cell proteins.^4^ However, the exact role of GRA8 in regulating host immune responses remains unclear.

The maintenance and regulation of cellular metabolism are important in cell fate-controlling diseases. Sepsis, defined as a systemic inflammatory response syndrome to infection, has significant health and economic effects associated with high mortality and morbidity.^5^ Mitochondrial dysfunction occurs early in sepsis. The severity of systemic inflammatory response syndrome and recovery of mitochondrial function are associated with survival.^6, 7, 8^ Therefore, understanding how specific metabolism regulators are altered in sepsis would help develop therapies. Mitochondria-targeted therapy is considered a promising strategy for preventing, mitigating or reversing systemic inflammatory response syndrome and for reducing sepsis. This intervention, known as ‘metabolic resuscitation,’ can improve mitochondrial activity via pharmacological or nutritional agents.^8, 9, 10^ However, the *T. gondii* GRA8-mediated mitochondrial and metabolic changes due to sepsis remain unexplained.

Mitochondria play a key role in the adaptive response by modulating their adenosine triphosphate (ATP)-generating capacity through oxidative phosphorylation (OXPHOS).^11, 12^ Mitochondrial diseases are mostly caused by impairment of the OXPHOS system or other energy metabolism defects.^6, 11, 12, 13^ OXPHOS enzymes have two genetic origins; therefore, protein synthesis is necessary in the cytoplasm and mitochondrion for complete OXPHOS function and adaptation to metabolic challenges.^12^ The role of sirtuins (SIRT1–7) in the pathogenesis of sepsis is a new area of mitochondria research.^6, 9, 10, 14^ SIRT3 is a major mitochondrial NAD^+^-dependent deacetylase that targets mitochondrial proteins for lysine deacetylation, thereby affecting regulators of cytoplasmic protein synthesis as well as stoichiometry of nuclear- and mitochondrial-encoded OXPHOS proteins to regulate cellular functions.^6, 12^ SIRT3 is a key regulator of the mitochondrial adaptive response to stress, including metabolic reprograming and antioxidant defense mechanisms. The sequential actions of nuclear SIRT1 and RELB induce SIRT3 expression and increase mitochondrial biogenesis during sepsis adaptation.^6^ Thus, the cellular mechanisms that control mitochondrial OXPHOS gene expression could be exploited in therapeutic approaches against sepsis.^15, 16^ In this study, we investigated the protective role of SIRT3 in septic shock to facilitate therapeutic drug discovery. Targeting GRA8 for mitochondrial metabolic reprogramming could be a therapeutic strategy for treating mitochondrial diseases.

We found that *T. gondii* GRA8 interacts with ATP5A1–SIRT3 signaling pathways to contribute to anti-septic activities *in vivo*. Thus, *T. gondii* GRA8-induced mitochondrial metabolic resuscitation may be exploited in therapeutic interventions against sepsis.

## Materials and methods

### Ethics statement

All animal experimental procedures were reviewed and approved by the institutional animal care and use committee of Hanyang University (protocol 2017-0025). All animal experiments were performed in accordance with Korean Food and Drug Administration guidelines.

### Mouse model of sepsis

Cecal ligation and puncture (CLP)-induced sepsis mouse model was prepared using 6-week-old C57BL/6 female mice (Samtako Bio, Gyeonggi-do, Korea), as previously described.^5^ Briefly, mice were anesthetized with pentothal sodium (50 mg kg^−1^, intraperitoneally), and a small abdominal midline incision was made to expose the cecum. The cecum was then ligated below the ileocecal valve, punctured twice through both surfaces, using a 22-gauge needle, and the abdomen was closed. The survival rate was monitored daily for 7 days. All animals were maintained in a pathogen-free environment.

### Mice and cell culture

Wild-type (WT) C57BL/6 mice were purchased from Orient Bio (Gyeonggi-do, Korea). PKCα^−/−^ (B6;129-*Prkca*^*tm1Jmk*^/J, 009068) and SIRT3^−/−^ (B6.129S6(Cg)-*Sirt3*^*tm1.1Fwa*^/J, 027975) mice were obtained from the Jackson Laboratory (Bar Harbor, MA, USA). All animals were maintained in a specific pathogen-free environment. HEK293T cells (ATCC-11268; American Type Culture Collection, Manassas, VA, USA) and HCT116 (ATCC-CCL247) were maintained in Dulbecco’s modified Eagle’s medium (Invitrogen, Waltham, MA, USA) containing 10% fetal bovine serum (Invitrogen), sodium pyruvate, nonessential amino acids, penicillin G (100 IU ml^−1^) and streptomycin (100 μg ml^−1^). Human monocytic THP-1 (ATCC TIB-202) cells were grown in RPMI-1640/glutamax supplemented with 10% fetal bovine serum and treated with 20 nM phorbyl myristate acetate (Sigma-Aldrich, St Louis, MO, USA) for 24 h to induce their differentiation into macrophage-like cells, followed by washing three times with phosphate-buffered saline. Primary bone marrow-derived macrophages (BMDMs) were isolated from C57BL/6 mice and cultured in Dulbecco’s modified Eagle’s medium for 3–5 days in the presence of macrophage colony-stimulating factor (R&D Systems, Minneapolis, MN, USA), as described previously.^2^ Transient transfections were performed using Lipofectamine 2000 (Invitrogen) or calcium phosphate (Clontech, Mountain View, CA, USA), according to the manufacturer’s instructions.

### Recombinant GRA8 protein

To obtain *T. gondii* ME49 strain purified GRA8 (GenBank accession no. XP_002369526.1) protein, GRA8 amino acid residues WT (1 to 269), T220A and N (23 to 224) were cloned with an N-terminal 6XHis tag into the pRSFDuet-1 Vector (Novagen, Madison, WI, USA) and induced, harvested and purified from *Escherichia coli* expression strain BL21(DE3)pLysS as described previously^2, 17^ in accordance with the standard protocols recommended by Novagen. rGRA8 was dialyzed with permeable cellulose membrane and tested for lipopolysaccharide contamination with a *Limulus* amebocyte lysate assay (BioWhittaker, Basel, Switzerland) and contained <20 pg ml^−1^ at the concentrations of rGRA7 protein used in the experiments described here.

### Protein purification and mass spectrometry

To identify GRA8-binding proteins, THP-1 cells expressing Flag-GRA8 or vector were harvested and lysed with NP-40 buffer (50 mM HEPES, pH 7.4,150 mM NaCl, 1 mM EDTA, 1% (v/v) NP40) supplemented with a complete protease inhibitor cocktail (Roche, Basel, Switzerland). Postcentrifuged supernatants were precleared with protein A/G beads at 4 °C for 2 h. Precleared lysates were mixed with αFlag antibody-conjugated with agarose beads for 4 h at 4 °C. Precipitates were washed extensively with lysis buffer. Proteins bound to beads were eluted and separated on a Nupage 4–12% Bis-Tris gradient gel (Invitrogen). After silver staining (Invitrogen), specific protein bands were excised and analyzed by ion-trap mass spectrometry at the Korea Basic Science Institute (Daejeon, Korea) Mass Spectrometry facility, and amino acid sequences were determined by tandem mass spectrometry and database searches.

### Reagents and antibodies

Cycloheximide (01810) and calf-intestinal alkaline phosphatase (CIP, P4978) were purchased from Sigma. Dimethyl sulfoxide (D8418, Sigma-Aldrich, St Louis, MO, USA) was added to the cultures at 0.1% (v/v) as a solvent control. Specific antibodies against ATP5C1 (PA5-29975) and NDUFA9 (459100) were purchased from Invitrogen. Antibodies specific for ATP5A1 (51), SIRT3 (14.45), VDAC (B-6), SDHA (B-1), UQCRC2 (G-10), COX IV (D-20), PGC-1α (H-300), PGC-1β (E-9), NRF1 (H-285), NRF2 (C-20), Tfam (H-203), SIRT1 (H-300), PKCα (C-20), actin (I-19), V5 (H-9), Flag (D-8), His (AD1.1.10) and GST (B-14) were purchased from Santa Cruz Biotechnology (Dallas, TX, USA).

### Plasmid construction

The plasmids encoding full length of the GRA8 (glutathione *S*-transferase (GST) or Flag-GRA8) and ATP5A1 (V5 or Flag-ATP5A1) plasmid have been previously described.^2, 18^ Plasmids encoding different regions and point mutants of GRA8 or ATP5A1 were generated by PCR amplification from full-length GRA8 or ATP5A1 complementary DNA and subcloning into a pEBG derivative encoding an N-terminal GST epitope tag or pcDNA3 vector between the *Bam*HI and *Not*I sites. All constructs for transient and stable expression in mammalian cells were derived from the pEBG-GST mammalian fusion vector and the pcDNA3-Neo expression vector. All constructs were sequenced using an ABI PRISM 377 automatic DNA sequencer (Foster City, CA, USA) to verify 100% correspondence with the original sequence.

### Complex V activity assay

The activity of complex V was determined using the MitoTox Complex V OXPHOS Activity Microplate Assay kit from Abcam (ab109907, Cambridge, MA, USA), and the manufacturer’s instructions were followed. The activity of complex V was measured by monitoring the change in absorbance at 340 nm over a period of 1 h at 30 °C. Oligomycin (Sigma, O4876) was used as a positive control for the assay.

### Statistical analysis

All data were analyzed using Student’s *t*-test with Bonferroni adjustment or analysis of variance for multiple comparisons, and are presented as mean±s.d. Statistical analyses were conducted using the SPSS (Version 12.0) statistical software program (SPSS, Chicago, IL, USA). Differences were considered significant at *P*<0.05. For survival, data were graphed and analyzed by the product limit method of Kaplan and Meier, using the log-rank (Mantel–Cox) test for comparisons using GraphPad Prism (version 5.0, La Jolla, CA, USA).

## Results

### GRA8 interacts with ATP5A1

**Figure 1:**
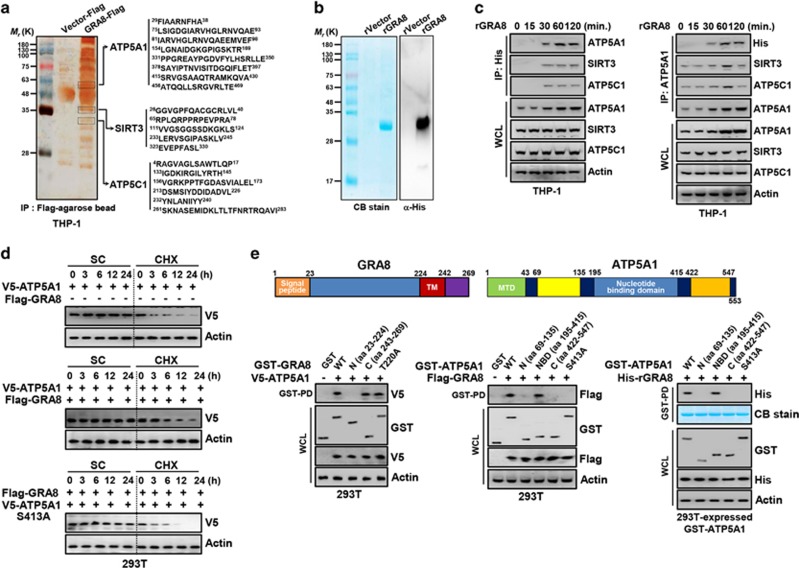
GRA8 interacts with ATP5A1 and SIRT3. (**a**) Identification of ATP5A1, SIRT3 and ATP5C1 by mass spectrometry analysis in THP-1 cell lysates expressed with GRA8 or vector. (**b**) Bacterially purified 6xHis-GRA8 were analyzed by Coomassie blue staining (left) or immunoblotting (IB) with αHis (right). (**c**) THP-1 cells were stimulated with rGRA8 (5 μg ml^−1^) for the indicated times, followed by immunoprecipitation (IP) with αHis-agarose bead or αATP5A1 and IB with αATP5A1, αSIRT3, αATP5C1, αHis and αActin. (**d**) GRA8-mediated increases of ATP5A1 stability. At 24 h after transfection with V5-ATP5A1and/or Flag-GRA8, 293T cells were treated with solvent control (SC) or cyclohexamide (CHX, 1 μg ml^−1^) for indicated times and cell lysates were used for IB with αV5 and αActin. (**e**) Binding mapping. Schematic diagrams of the structures of GRA8 and ATP5A1 (upper). At 48 h after transfection with mammalian glutathione *S*-transferase (GST) or GST-GRA8 and truncated mutant constructs together with V5-ATP5A1, or GST-ATP5A1 constructs together with Flag or His-GRA8, 293T cells were used for GST pull down, followed by IB with αV5, αFlag or αHis. Whole cell lysates (WCLs) were used for IB with αGST, αV5, αFlag, αHis or αActin. The data are representative of four independent experiments with similar results (**a**–**e**).

To establish a role for *T. gondii* GRA8 in treating mitochondrial diseases in macrophages, we investigated whether GRA8 interacts with molecules involved with mitochondrial metabolism. GRA8 complexes were subjected to co-immunoprecipitation with THP-1 cells containing vector or Flag-GRA8. The purified GRA8 complexes were identified by mass spectrometry analysis where they contained ATP synthase subunit-α (ATP5A1, 60K), SIRT3 (37K) and ATP synthase subunit γ (ATP5C1, 32K) (Figure 1a).

To examine the role of GRA8 in macrophages, we generated bacterially purified His-tagged rGRA8, as described previously.^2, 17^ The purified rGRA8 (30 kDa) was confirmed by SDS–polyacrylamide gel electrophoresis and immunoblotting (Figure 1b). No significant differences compared with vector controls were observed for rGRA8-induced cytotoxicity in macrophages (data not shown). Endogenous co-immunoprecipitation showed that GRA8 interacted strongly but temporarily (from 30 to 120 min) with endogenous ATP5A1, SIRT3 and ATP5C1 after stimulation with rGRA8 in THP-1 cells, and vice versa (Figure 1c). Remarkably, ATP5A1, SIRT3 and ATP5C1 bound directly and specifically to bacterially purified His-rGRA8 protein *in vitro* (Supplementary Figure S1). The exogenous expression of rGRA8 markedly increased the expression level of endogenous ATP5A1 (Figure 1c). Cycloheximide treatment confirmed that GRA8 expression significantly stabilized the ATP5A1 level (Figure 1d). A large-scale proteomics analysis of the human kinome^19^ and computational sequence analysis^20^ predicted that ATP5A1 S413 is a critical site for the activity and stability of the ATP5A1 protein. Remarkably, GRA8 also bound and stabilized the ATP5A1 S413A mutant (Figure 1d).

GRA8 contains a signal sequence, N-terminal domain (amino acids (aa) 23–224), transmembrane domain and C-terminal domain (aa 243–269) (Figure 1e).^19^ In 293T cells, detailed mapping using various mammalian GST-GRA8 or ATP5A1 fusions and truncated mutants of V5-ATP5A1 or Flag-GRA8 indicated that the C-terminal domain of GRA8 had minimal binding affinity with ATP5A1, but ATP5A1 with the nucleotide-binding domain (aa 195–415) bound GRA8 as strongly as ATP5A1 WT (Figure 1e). GST pull-down assays using truncated mutants of 293T-expressed GST-ATP5A1 and His-rGRA8 showed that the nucleotide-binding domain of ATP5A1 bound directly and specifically to His-rGRA8 *in vitro*. Furthermore, a GRA8 T220A mutant that interferes with protein kinase C (PKC) phosphorylation^19, 20^ did not affect the interaction between GRA8 and ATP5A1 (Figure 1e). Thus, GRA8 effectively enhances the stability of ATP5A1 in a binding-dependent manner.

### Mitochondrial targeting of GRA8 via PKCα phosphorylation

**Figure 2:**
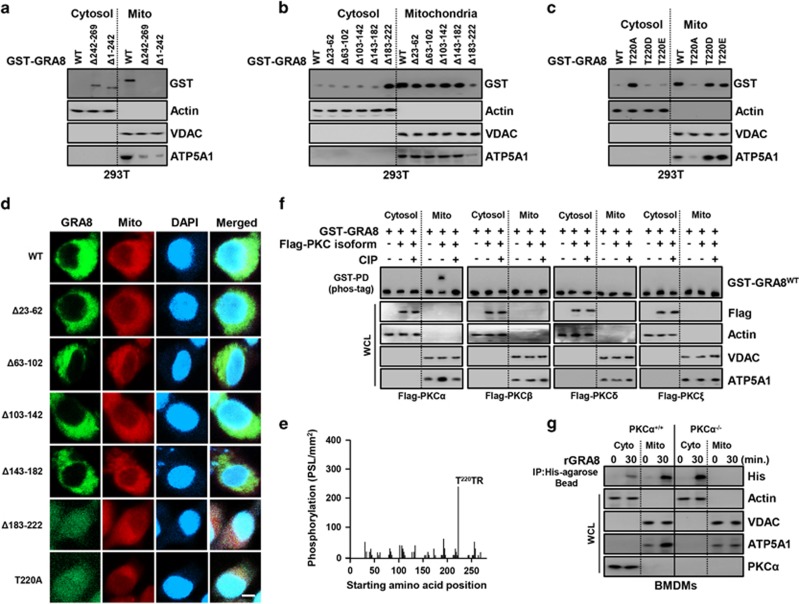
Protein kinase-Cα (PKCα)-dependent phosphorylation of GRA8 was essential for mitochondrial localization. (**a**–**c**) Subcellular fractionation of 293T cells stably expressing either GST-GRA8 WT, Δ23–62, Δ63–102, Δ103–142, Δ143–182, Δ183–222, Δ242–269, Δ1–242 or GST T220A/D/E. Mitochondrial and cytosolic fractions were fractionated and analyzed for expression of glutathione *S*-transferase (GST) by immunoblotting (IB). Purity of the fractions was assessed by blotting for VDAC (voltage-dependent anion channel; mitochondrial protein) and actin (cytosolic protein). (**d**) Representative immunofluorescence images of 293-GRA8-GFP cells expressing wild-type (WT) and deletion mutants were colocalized with MitotrackerDeep Red FM (100 nM). Scale bar, 20 μm. (**e**) Mapping of PKCα phosphorylation sites on GRA8 by tiled peptide array analysis using purified recombinant PKCα. Phosphorylation intensity of 15-amino acid peptides that span full-length GRA8 and are each shifted by 3 amino acids was detected using MultiGauge version 3.0. The threonine in the peptides that showed a phosphorylation signal stronger than 100 PSL mm^−2^ is indicated above the corresponding peaks. (**f**) Phos-tag and SDS–polyacrylamide gel electrophoresis (SDS-PAGE) analyses of GST-GRA8 together with Flag-tagged isoform of PKC in 293T cells left untreated (CIP−) or treated with calf-intestinal alkaline phosphatase (CIP+), and subcellular fractionation as the experimental conditions follow the same pattern in (**a**). (**g**) Bone marrow-derived macrophages (BMDMs) from PKCα^+/+^ and PKCα^−/−^ were stimulated with rGRA8 for 30 min, followed by subcellular fractionation as the experimental conditions follow the same pattern in (**a**). The data are representative of four independent experiments with similar results (**a**–**g**).

Given that GRA8 associates with mitochondrial proteins, we evaluated whether GRA8 tended to localize in mitochondria. We expressed GST-GRA8 in 293T cells via transient transfection and conducted subcellular fractionation of mitochondria. The N-terminal domain of GRA8 localized almost completely with mitochondria (Figure 2a). To determine whether the PSORT-predicted N-terminal mitochondrial presequence^21, 22^ in GRA8 is involved with recruitment to mitochondria, we generated N-terminal deletion mutants of GRA8 (Figure 2b). The N-terminal domain (aa 23–182) of GRA8 localized primarily to the cytosol. A Δ183–222 mutant and GRA8 point mutant (T220A) exhibited diminished mitochondrial localization and ATP5A1 stabilization, but both functions increased markedly in a phosphomimetic mutant (T220D and T220E) (Figures 2b and c). These results indicate that residues 183–222 constitute the minimum N-terminal sequence necessary for both GRA8 mitochondrial association and ATP5A1 stabilization. GRA8 is also likely to be T220 phosphorylation dependent. Consistent with these biochemical data, imaging of the GRA8 Δ183–222 mutant and T220A point mutant confirmed their diminished mitochondrial localization and ATP5A1 stabilization (Figure 2d and Supplementary Figure S2A).

Several strategies were used to confirm that GRA8 was phosphorylated by a PKC isoform. An *in vitro* phosphorylation assay was performed using purified recombinant PKCα and a nonbiased overlapping peptide array covering the entire GRA8 sequence.^19^ The GRA8 N-terminal peptide ^218^TTTTRNVLLRTAILAA^233^ had a phosphorylation signal >200 PSL mm^−2^, unlike the C terminus (Figure 2e). We performed Phos-tag gel electrophoresis using a Phos-tag biomolecule that specifically binds phosphorylated proteins and retards their migration in gels.^2^ GRA8 migrated more slowly and produced an ‘up-shifted’ band when co-expressed with PKCα, but not when co-expressed with PKCβ, PKCδ or PKCξ (Figure 2f). Interestingly, the GRA8 point mutation (T220A) markedly decreased phosphorylation in Phos-tag gel electrophoresis, but not the phosphomimetic mutant (T220D and T220E) (data not shown). Thus, PKCα can specifically phosphorylate T220 in GRA8, and hence GRA8 is a substrate of PKCα. Consistent with the results in Figures 2a–f, the mitochondrial localization of GRA8 and interaction with ATP5A1 decreased markedly in BMDMs from PKCα^–/–^ mice (Figure 2g and Supplementary Figure S2B), and in THP-1 cells subjected to knockdown with small hairpin RNA specific to PKCα and stimulated with rGRA8 (data not shown). Therefore, the PKCα-mediated phosphorylation of GRA8 at Thr220 (N terminus) is essential for the mitochondrial targeting of GRA8, and the ATP5A1 interactions (C terminus) are genetically separable.

### rGRA8 induces mitochondrial activity and biogenesis in a PKCα-dependent manner

**Figure 3:**
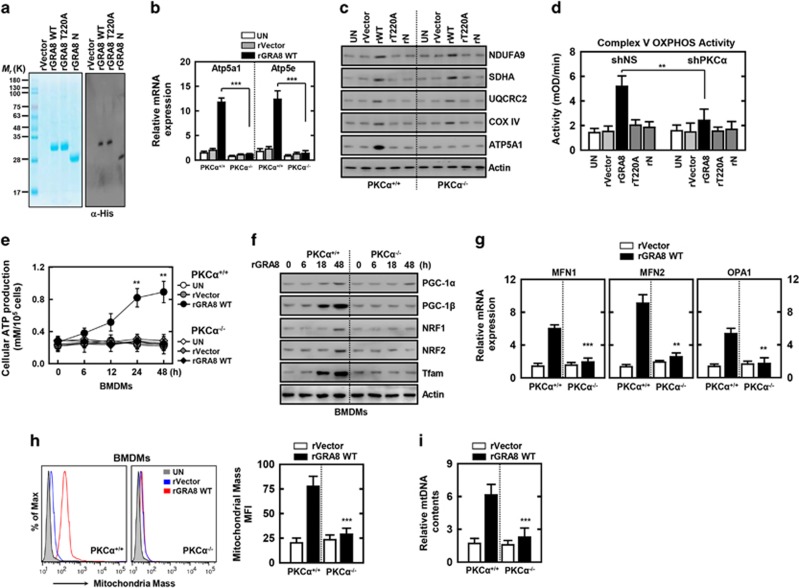
The rGRA8 treatment increases the induction of mitochondrial activity and biogenesis via protein kinase-Cα (PKCα). (**a**) Bacterially purified 6xHis-GRA8-WT and its mutants were analyzed by Coomassie blue staining (left) or immunoblotting (IB) with αHis (right). (**b–d**) Bone marrow-derived macrophages (BMDMs) from PKCα^+/+^ and PKCα^−/−^ (**b**, **c**) or THP-1 cells were transduced with lentivirus-shRNA-NS or lentivirus-shRNA-PKCα (multiplicity of infection (MOI)=100) with polybrene (8 μg ml^−1^) (right) for 2 days (**d**), the cells were stimulated with rGRA8 (1 μg ml^−1^) and its mutants for 6 h (**b**) and 24 h (**c**, **d**) and subjected to quantitative real-time PCR (**b**), IB (**c**) or enzymatic activity (**d**) of oxidative phosphorylation (OXPHOS) genes. (**e**–**g**) BMDMs from PKCα^+/+^ and PKCα^−/−^ were stimulated with rGRA8 for the indicated times and subjected to cellular adenosine triphosphate (ATP) production (**e**), IB analysis with αPGC-1, αNRF1, αNRF2, αTfam and αActin (**f**) or quantitative real-time PCR of fusion genes (**g**). (**h**) Mitotracker fluorescence signals assessed by a flow cytometric analysis. (Left) Representative histograms from seven independent replicates. (Right) Bar graph indicates the mitochondrial mass mean fluorescence intensities (MFIs). Results are expressed as means±s.d. of seven experiments. (**i**) Mitochondrial DNA (mtDNA) content in BMDMs measured by quantitative real-time PCR. The mtDNA content was normalized to nuclear DNA. Significant differences (**P<0.01; ***P<0.001) compared with PKCα^+/+^ or shRNA-NS (Non-specific) (**b**, **d**, **e** and **g**–**i**).The data are representative of five independent experiments with similar results (**a**–**g** and **i**).

To examine the role of the GRA8 N terminus in mitochondrial metabolism,^13, 23^ we generated bacterially purified His-tagged rGRA8 mutant proteins and confirmed them using SDS–polyacrylamide gel electrophoresis and immunoblotting (Figure 3a). No significant differences compared with the vector controls were observed for rGRA8 mutant-induced cytotoxicity in macrophages (Supplementary Figure S3A).

Mitochondrial oxidative metabolism modulates the energetic demands of cells via OXPHOS.^6, 18, 23^ To determine the role of rGRA8 in mitochondria, we examined the mRNA and protein expression levels for macrophage mitochondrial respiratory chain complexes I–V. Complex V genes (ATP5A1 and ATP5E) and proteins, and the OXPHOS activity were upregulated significantly by rGRA8 N-terminus treatment (compared with other mutants) in a PKCα-dependent manner (Figures 3b–d and Supplementary Figure S3B). The mitochondrial ATP levels were also increased significantly by rGRA8 N-terminus treatment (Figure 3e and Supplementary Figure S3C). We next investigated whether rGRA8 treatment of BMDMs led to the induction of genes and proteins regulating mitochondrial biogenesis and mass. Indeed, rGRA8 treatment of BMDMs robustly upregulated the expression of mRNA and proteins involved with mitochondrial biogenesis (PGC-1β and Tfam) and fusion (MFN1, MFN2 and OPA1) via PKCα (Figures 3f and g and Supplementary Figures S3DE). Similarly, we observed marked increases in the mitochondrial mass and DNA content of rGRA8-treated macrophages, but significant decrease in the mitochondrial mass and DNA content of rGRA8-treated PKCα^−/−^ BMDMs (Figures 3h and i), but the rGRA8-induced mitochondrial function, activity and biogenesis were independent of mitochondrial ROS generation (Supplementary Figure S3F). Thus, rGRA8 treatment enhanced mitochondrial OXPHOS, biogenesis, mass and ATP synthesis via PKCα in macrophages.

### rGRA8 induces the deacetylation of ATP5A1 (K506 and K531) by SIRT3

GRA8 associates with mitochondrial SIRT3 (Figure 1) that regulates homeostasis and numerous metabolic processes (for example, fatty acid oxidation, OXPHOS and the tricarboxylic acid cycle), and hence new targets and substrates for SIRT3-dependent lysine deacetylation have been identified.^12, 23, 24^ SIRT3 interacts with ATP5A1 and regulates its acetylation status and activity.^25^

**Figure 4:**
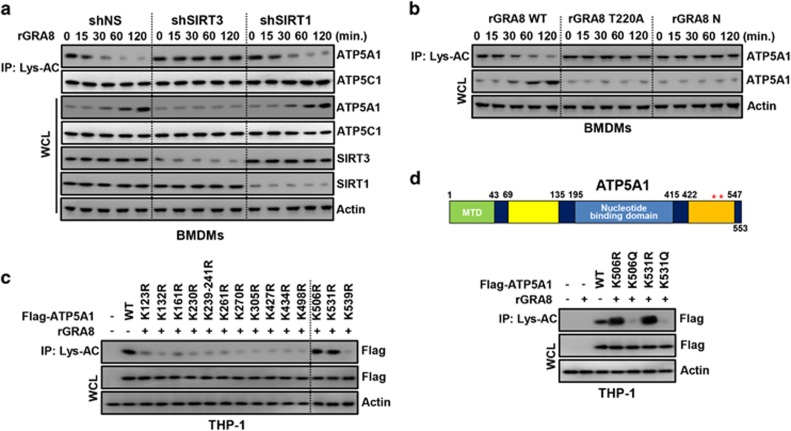
The rGRA8 treatment increases the induction of deacetylation of ATP5A1 via SIRT3. Bone marrow-derived macrophages (BMDMs) were transduced with lentivirus-shRNA-NS (Non-specific) or lentivirus-shRNA-SIRT1 or SIRT3 (multiplicity of infection (MOI)=100) with polybrene (8 μg ml^−1^) (right) for 2 days (**a**), BMDMs (**b**) or THP-1 cells stably expressing either ATP5A1 or its mutants (**c**, **d**), the cells were stimulated with rGRA8 (1 μg ml^−1^) and its mutants for the indicated times and subjected to immunoprecipitation (IP) with αLys-AC and immunoblotting (IB) with αATP5A1, αATP5C1, αSIRT1, αSIRT3, αFlag and αActin. Schematic diagrams of the structures of ATP5A1 (**d**, upper).The data are representative of five independent experiments with similar results.

We examined the acetylation levels of OXPHOS V proteins under rGRA8 treatment. First, we confirmed the acetylation of ATP5A1 and ATP5C1 in BMDMs using an anti-acetyl-lysine antibody to immunoprecipitate endogenous ATP5A1 and ATP5C1. The acetylation level of ATP5A1 was reduced in a time-dependent manner by WT rGRA8 but not ATP5C1, and the acetylation status of ATP5A1 was regulated by mitochondrial SIRT3 but not by cytosolic SIRT1 (Figure 4a). The T220A (mitochondria non-targeting) mutant and rGRA8 N terminus (ATP5A1 binding deficient) failed to significantly change the ATP5A1 acetylation level compared with WT rGRA8 (Figure 4b).

To determine how specific residues affected ATP5A1 acetylation, we created substitution mutations at positions predicted to mimic acetylated lysine (K→Q) or nonacetylated lysine (K→R).^26, 27^ Figures 4c and d show that the acetylation levels of the K506R and K531R mutants were similar to that of WT ATP5A1, and the acetyl-mimetic mutants (K506Q and K531Q) exhibited markedly increased deacetylation compared with WT ATP5A1. Thus, GRA8 interacts with SIRT3 in the mitochondria to induce the acetylation of ATP5A1 (K506 and K531).

### rGRA8 protects mice from systemic sepsis

**Figure 5:**
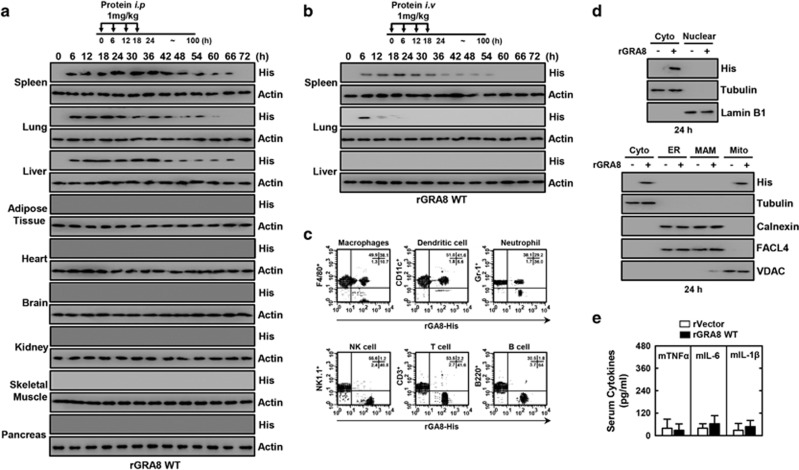
Therapeutic rGRA8 proteins are uptaken by cells of the reticuloendothelial system in mice. (**a**, **b**) Schematic of the pharmacokinetic analysis in mice treated with rGRA8 (upper). Pharmacokinetic analysis of proteins in the various organs, followed by immunoblotting (IB) with αHis and αActin. The data are representative of three independent experiments with similar results. Fluorescence-activated cell sorting (FACS) analysis in spleen (**c**), tissue subcellular fractionation in spleen (**d**), and (**e**) serum cytokine levels were evaluated 24 h after being injected with rGRA8 (10 mice per group). Significant differences compared with rVector-treated mice.

**Figure 6:**
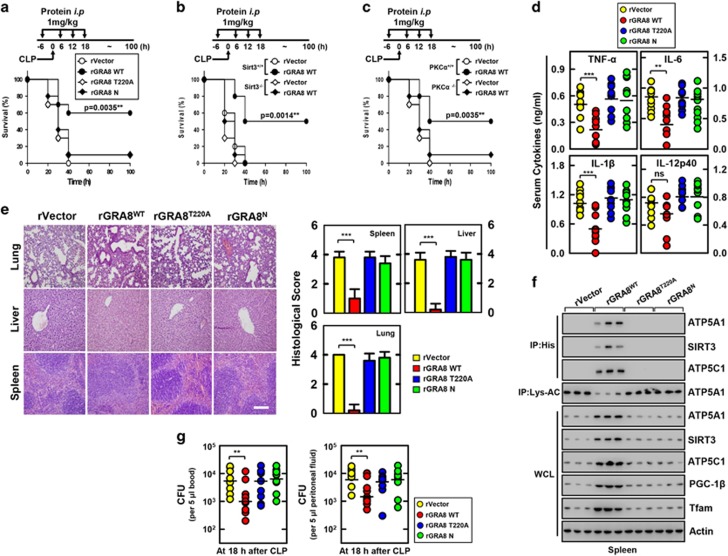
The rGRA8 protects mice from cecal ligation and puncture (CLP)-induced polymicrobial sepsis. (**a**–**c**) Schematic of the CLP model treated with rGRA8 or its mutants (upper). The survival of mice was monitored for 7 days; mortality was measured for *n*=25 mice per group (lower). Statistical differences compared with the rVector-treated mice are indicated (log-rank test). The data are representative of two independent experiments with similar results. (**d**) Serum cytokine levels and (**e**) representative hematoxylin and eosin (H&E) staining of the lung, liver and spleen (left) from 10 mice per group. Histopathology scores were obtained from H&E stained as described in Methods (right) were determined at 30 h in CLP mice were treated with rGRA8 or its mutants. Scale bar, 200 μm. (**f**) Splenocytes were used for immunoprecipitation (IP) with αHis or αLys-AC, followed by immunoblotting (IB) with αATP5A1, αATP5C1 or αSIRT3. Whole cell lysates (WCLs) were used for IB with αATP5A1, αATP5C1, αSIRT3, αPGC-1, αTfam or αActin. The data are representative of three independent experiments with similar results. (**g**) The bacterial burden was evaluated 18 h after treatment of CLP mice with rGRA8 or its mutants (*n*=10 mice per group). Results are expressed as means±s.d. (10 mice per group (**d**, **g**). Significant differences (**P<0.01; ***P<0.001) compared with rVector-treated mice (**d**, **e** right, and **g**).

We aimed to determine whether rGRA8 protects mice from septic shock due to polymicrobial peritonitis using a murine CLP model of polymicrobial infection that triggers systemic inflammatory response syndrome and is typically fatal in humans.^5^ As shown in Figure 5, rGRA8 proteins are uptaken by cells of the reticuloendothelial system in the spleen, lung and liver (Figures 5a–c). The pharmacokinetics of rGRA7 proteins localized in mitochondria was maintained for up to 36 h and gradually cleared until 60–66 h (Figure 5d). However, no significant difference was observed for inflammation in blood (Figure 5e). First, we tested the preventative (pretreatment) and protective (after treatment) efficacy of rGRA8 and its mutants against CLP-induced mortality in mice. WT rGRA8 prevented and protected against CLP-induced death in 30% and 20% of mice, respectively (Supplementary Figures S4A and B). Pre- and posttreatment of CLP mice with WT rGRA8 obtained dose-dependent protection, where 60% of the mice were protected from CLP-induced death when treated with WT rGRA8 at 1 mg kg^−1^ per mouse (Figure 6a), and this protection depended on SIRT3 and PKCα (Figures 6b and c). The serum concentrations of the proinflammatory cytokines tumor necrosis factor-α, interleukin (IL)-6, IL-1β and IL-12p40 were attenuated significantly in WT rGRA8-treated mice, but the IL-10 and IL-4 concentrations did not change significantly (Figure 6d and Supplementary Figure S4C). These changes were accompanied by reduced infiltration of immune cells and decreased damage to the lung, liver and spleen according to hematoxylin and eosin staining (Figure 6e).

We tested whether rGRA8 was pharmacologically active *in vivo*. The *in vivo* detection of mitochondria-related target protein binding profiles and OXPHOS activity might be important for evaluating new compounds when searching for therapeutic drugs to treat lethal inflammatory disease. Consistent with the *in vitro* data (Figures 1c, 3f and 4), treatment with WT rGRA8 markedly increased the binding of rGRA8 to ATP5A1, SIRT3 and ATP5C1. The ATP5A1 deacetylation level and mitochondrial biogenesis protein levels were also increased in splenocytes (Figure 6f). We investigated whether WT rGRA8 enhances bacterial clearance because CLP-induced lethality is positively correlated with bacterial colony counts in peripheral blood and peritoneal fluid,^5^ and WT rGRA8 enhances mitochondrial activity in macrophages. Treating CLP mice with WT rGRA8 dramatically decreased the bacterial colony counts in peritoneal fluid and blood (Figure 6g). Furthermore, the survival rates increased and the bacterial loads decreased after infection with *Escherichia coli*, *Staphylococcus aureus* or *Pseudomonas aeruginosa*, but there were no significant differences in these bacteria when grown in BHI broth with or without rGRA8 (Supplementary Figure S5). Therefore, rGRA8-mediated mitochondrial metabolic resuscitation has therapeutic potential to ameliorate CLP-induced sepsis.

## Discussion

**Figure 7:**
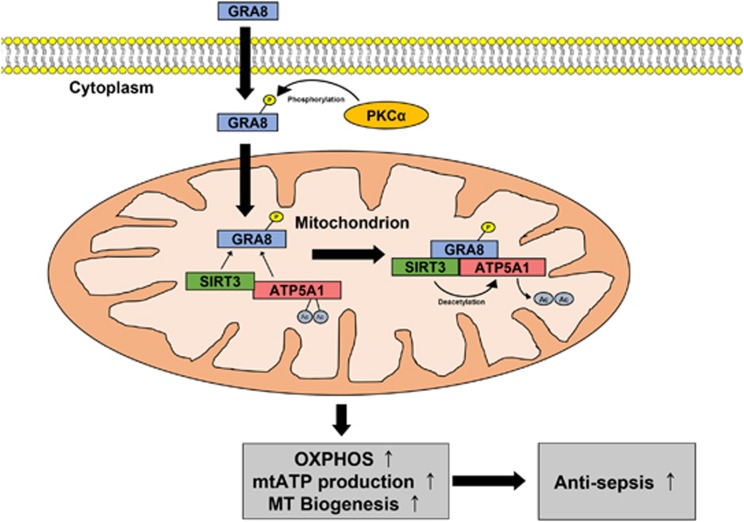
Schematic model for the roles of GRA8 and GRA8-mediated regulatory pathways against sepsis. Please see the Discussion for detail.

This study identified a new anti-sepsis therapeutic strategy based on *T. gondii* GRA8-induced mitochondrial metabolic resuscitation that represents a potential paradigm shift as an urgently needed therapeutic intervention (Figure 7). We found that: (1) the C terminus of *T. gondii* GRA8 interacts directly with the S413 residue of ATP5A1 to achieve stabilization; (2) GRA8 has a mitochondria-targeting sequence (aa 183–222), and PKCα-mediated phosphorylation of *T. gondii* GRA8 (Thr220) is required for mitochondrial targeting and interaction with ATP5A1 and SIRT3 in mitochondria; (3) the mitochondrial-targeting action and ATP5A1 binding action of GRA8 are functionally and genetically separable; (4) GRA8 interacts with SIRT3 to facilitate the ATP5A1 (K506 and K531) deacetylation-mediated mitochondrial biogenesis and activity via PKCα (5) rGRA8 protects mice from sepsis caused by polymicrobial infection via metabolic resuscitation; and (6) rGRA8 enhances bacterial clearance and it could be a new therapy for septic shock and other microbe-mediated diseases. Collectively, these observations suggested that rGRA8-mediated mitochondrial metabolic resuscitation can provide a unique opportunity to urgently treat life-threatening septic shock.

Evidence suggests that host–pathogen interactions facilitate the coevolution of the toxoplasmosis-causing pathogen *T. gondii* with its host.^2, 4, 17^ According to a yeast two-hybrid analysis using GRA8 as the bait in a HeLa cell complementary DNA library, GRA8 binds to the following metabolism-related proteins: nuclear gene encoding mitochondrial protein, transcript variant 1 (NM_004493, NM_005175); ATP synthase; H^+^-transporting mitochondrial F0 complex subunit C1 (subunit 9) (ATP5G1); phosphoglycerate dehydrogenase (PHGDH); pyruvate kinase, muscle transcript variant 3 (PKM2); and cytochrome b5 reductase 3, transcript variant M (CYB5R3).^4^ Our results agreed with previous studies and we also showed that GRA8 binds to mitochondrial proteins ATP5A1, ATP5C1 and SIRT3. Furthermore, GRA8-induced mitochondrial metabolic activation and biogenesis are crucial for modulating mitochondrial diseases *in vitro* and *in vivo*. We found that GRA8 interacted with a number of host cell proteins, including OXPHOS proteins and mitochondrial enzymes, thereby indicating a new role for GRA8 in the regulation of mitochondria.

Sepsis is associated with the aerobic glycolysis of glucose (Warburg effect) and ATP consumption promotes sepsis metabolism.^6, 14, 15, 16, 28^ Thus, inhibiting glycolysis and activating the OXPHOS capacity may contribute to the anti-sepsis effect in macrophages. Understanding the role of GRA8 in these mechanisms might provide a new basis for sepsis treatment, thereby helping to overcome resistance to chemotherapy or radiotherapy.

Some clinical trials and many experimental studies have reported improved mitochondrial activity and positive effects on outcomes driven by pharmacological and nutritional management strategies, thereby suggesting the need for further research. Micronutrients can regulate sepsis processes as metabolic resuscitators. Thus, thiamin (vitamin B1) is a water-soluble vitamin with an essential role in cellular energy metabolism and as a cofactor in the multi-enzyme pyruvate dehydrogenase complex, and it is essential for converting pyruvate from glucose into acetyl-CoA before entering the tricarboxylic acid cycle, with subsequent OXPHOS activity and ATP generation.^29, 30^ Experimental studies indicate that ascorbic acid (vitamin C), tocopherol (vitamin E), selenium and zinc can act as mitochondrial antioxidants.^31, 32, 33, 34^ Potential interventions exist to restore mitochondrial function during sepsis but their clinical benefits are unproven;^31, 35^ for example, coenzyme Q10 is a component of the mitochondrial electron transport chain,^36^ L-carnitine is essential for mitochondrial fatty acid β-oxidation.^37^ Cytochrome oxidase is the terminal oxidase of the electron transport chain,^38^ and melatonin and its metabolites with potent antioxidant properties accumulate in mitochondria.^39^ SIRT3 can regulate certain cancer-related processes and hence it is a potential therapeutic target in cancer treatment.^40^ Class III histone deacetylase activators of SIRT3 (such as resveratrol) are in the early stages of clinical trials and under testing for safety in the treatment of various cancers.^41, 42^ The Chinese medicinal plant *Scutellaria baicalensis* Georgi is a potential anticancer drug because it contains oroxylin A that inhibits glycolysis and the binding of HK II to mitochondria (which depends on SIRT3) in human breast carcinoma cells.^43^ A novel SIRT3 inhibitor called 5-amino-2-phenyl-benzoxazole is a good starting point for the future development of SIRT3 inhibitors with novel structural scaffolds.^44^ Understanding the anti-sepsis of SIRT3 will help to develop novel therapeutic strategies.

The rGRA8 protein has potential therapeutic uses but it does not satisfy the direct requirements for anti-sepsis agents as feasible alternatives to conventional chemotherapy. Concerns regarding GRA8 include its uncertain specificity and selectivity, inherent limitations of the animal models used for its study, unknown off-target effects, incomplete pharmacokinetics, limited safety data and the unclear feasibility of *in vivo* proof-of-concept studies. Further analyses are required to determine the suitability of rGRA8 for *in vivo* use in patients with sepsis and colon cancer.

We provided evidence of a critical role for PKCα in mediating the phosphorylation of *T. gondii* GRA8 that facilitates interactions between GRA8 and ATP5A1 or SIRT3, thereby contributing to metabolic resuscitation against sepsis and cancer (Figure 7). The N and C termini of GRA8 induce metabolic protective effects against sepsis *in vivo*. Thus, GRA8 has a new role in regulating mitochondrial activity to achieve protective immunity in the host. We provided a proof of concept for host-directed therapeutic strategies that manipulate GRA8-mediated host metabolic networks. Further research is needed to develop effective GRA8-based therapeutic targets and to understand how GRA8 regulates host defense strategies against sepsis, cancer and other mitochondria-related infectious diseases.
