# Supplementary material for: Toxoplasma gondii GRA8 induces ATP5A1–SIRT3-mediated mitochondrial metabolic resuscitation: a potential therapy for sepsis
Source: Exp Mol Med. 2018 Mar 30;50(3):e464–. doi: 10.1038/emm.2017.308 (PMC5898902; doi:10.1038/emm.2017.308)
Supplement: Supplementary Material [file emm2017308x1.docx]

**SUPPLEMENTARY FIGURE and FIGURE LEGENDS**

**
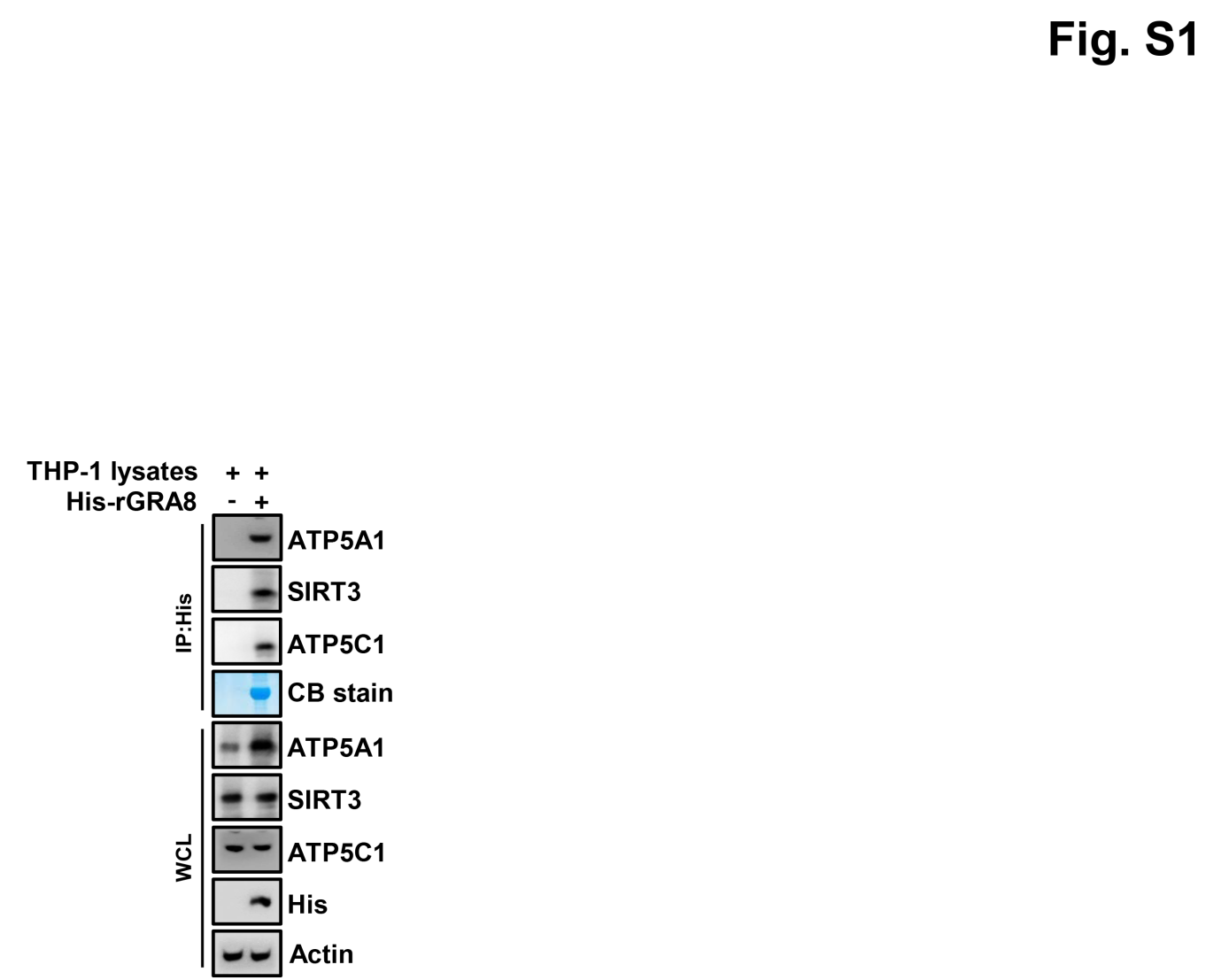
**

**Figure 1. GRA8 directly interacts with ATP5A1 and SIRT3.**

THP-1 cell lysates incubated with a His-tagged rGRA8 (2 μg), followed by immunoprecipitation (IP) with αHis-agarose bead and IB with αATP5A1, αSIRT3, αATP5C1, αHis, and αActin. CB stain, staining of His-rGRA8 with Coomassie blue. The data are representative of four independent experiments with similar results.

**
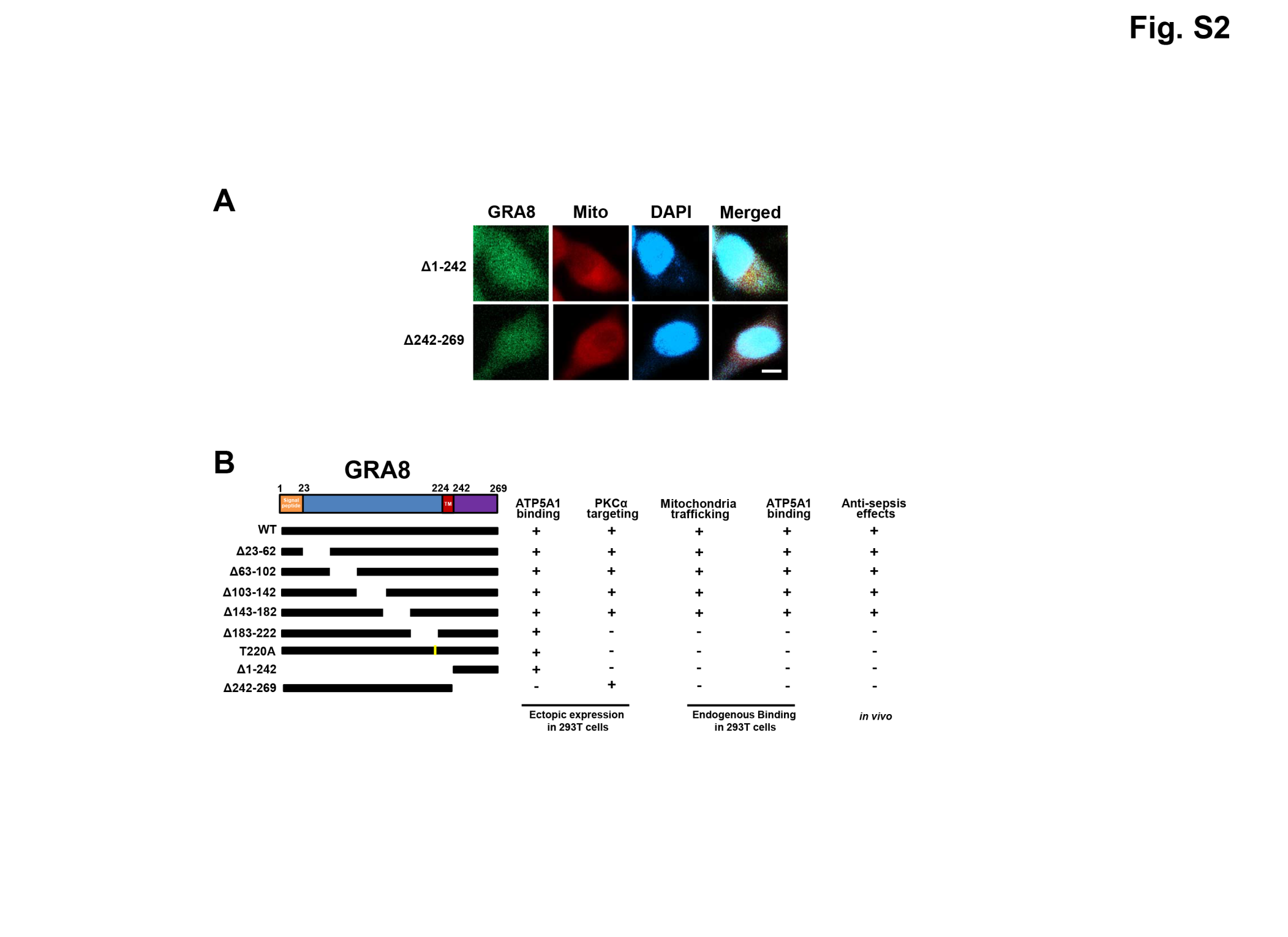
**

**Figure 2. The summary of GRA8 in mitochondria.**

(**A**) Representative immunofluorescence images of 293-GRA8-GFP cells expressing deletion mutants were colocalized with Mitotracker®Deep Red FM (100 nM). Scale bar : 20 μm. The data are representative of four independent experiments with similar results. (**B**) Summary of the interactions of GRA8 WT and its mutants with ATP5A1, mitochondria trafficking, and biological relevance with sepsis.


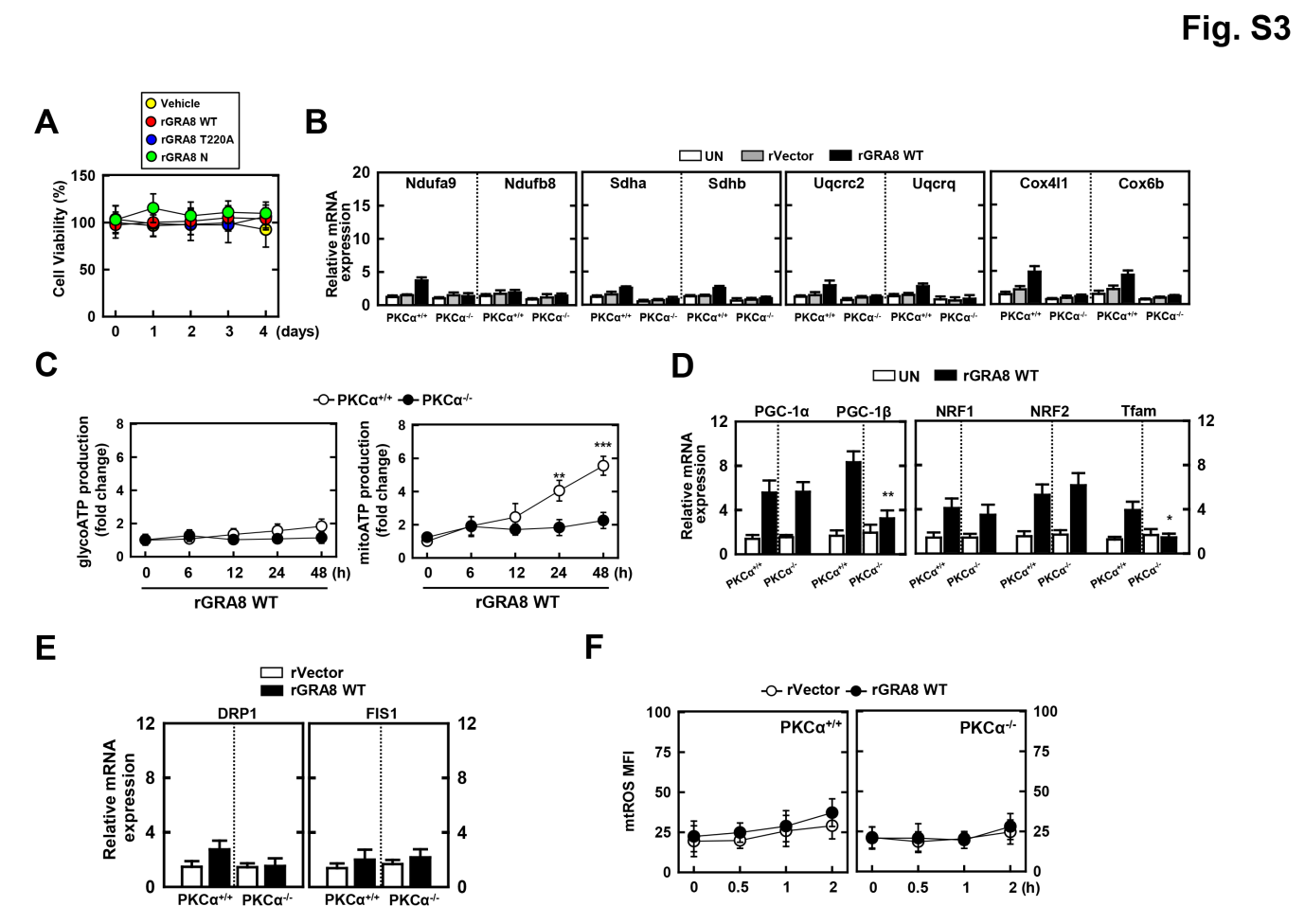


**Figure 3. The effects of rGRA8 in mitochondria via PKCα.**

(**A**) BMDMs were incubated with rGRA8 (1 μg/ml) and its mutants for the indicated times and then cell viability measured with MTT assay. (**B**-**F**) BMDMs from PKCα^+/+^ and PKCα^-/-^, the cells were stimulated with rGRA8 (1 μg/ml) and its mutants for the 6h (**B**, **D**, and **E**) or for the indicated times (**C** and **F**) and subjected to quantitative real-time PCR of OXPHOS genes (**B**), ATP measurement for glycolysis or mitochondria (**C**), quantitative real-time PCR of biogenesis genes (**D**) or fission genes (**E**), or analyzed for mitochondrial ROS (**F**). Significant differences (**P* < 0.05;***P* < 0.01; ****P* < 0.001) compared with PKCα^+/+^ (**C**, **D**, and **F**).The data are representative of five independent experiments with similar results (**A**-**F**).

**
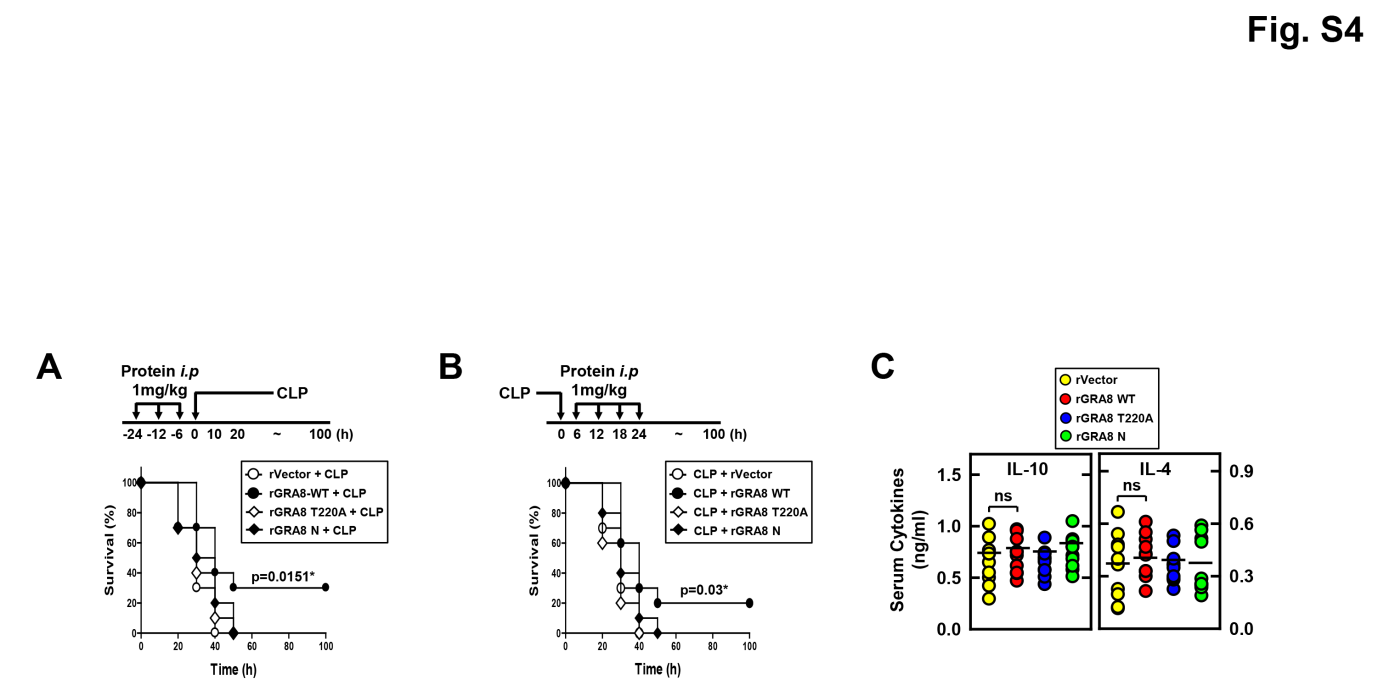
**

**Figure 4. The effects of rGRA8 from CLP-induced polymicrobial sepsis.**

(**A** and **B**) Schematic of the CLP model treated with rGRA8 or its mutants (upper). The survival of mice was monitored for 7 days; mortality was measured for n=10 mice per group (lower). Statistical differences compared to the rVector-treated mice are indicated (log-rank test). The data are representative of two independent experiments with similar results. (**C**) Serum cytokine levels (10 mice per group). Significant differences compared with rVector-treated mice. Ns, non-significant.

**

**

**Figure 5. The rGRA8 protects mice from CLP-induced polymicrobial sepsis.**

(**A**, **D**, and **G**) Schematic of the bacteria infection model treated with rGRA8 or its mutants (upper). The survival of mice was monitored for 7 or 14 days; mortality was measured for n=10 mice per group (lower). Statistical differences compared to the rVector-treated mice are indicated (log-rank test). The data are representative of two independent experiments with similar results. (**B**, **E**, and **H**) The bacteria burden was evaluated 24 h or 2 days after infected with bacteria with rGRA8 or its mutants (n = 10 mice per group). Significant differences (**P* < 0.05;***P* < 0.01; ****P* < 0.001) compared with rVector-treated mice. (**C**, **F**, and **I**) Bacteria were cultures in LB broth contained in presence of rVector, rGRA8 or its mutants (50 μg/ml) for the indicated times at 37℃. Measure the OD_600_ every 6 h. Data shown are the means ± SD of three experiments.

**SUPPLEMENTARY INFORMATION**

**Bacteria strains**

*Escherichia coli* (Serotype O86:K61 (B7), ATCC 12701)*, Staphylococcus aureus* (ATCC 6538), and *Pseudomonas aeruginosa* (ATCC 10145) were grown at 37 °C in brain-heart–infusion (BHI) broth medium (BD). For all assays, mid-log-phase bacteria (absorbance, 0.5) were used. Batch cultures were aliquoted and stored at −80 °C. Representative vials of bacteria were thawed and enumerated for viable colony-forming unit (CFU) on BHI agar (BD). The effective concentration of LPS was <50 pg/ml in those experiments with a bacterium-to-cell ratio of 10:1. All infection experimental procedures were reviewed and approved by the Institutional Biosafety Committees of Hanyang University (protocol 2014-01).

**GST pulldown, immunoblot, and immunoprecipitation analysis**

GST pulldown**,** immunoprecipitation, and immunoblot assays were performed as described previously ^1,2^. For GST pulldown, cells were harvested and lysed in NP-40 buffer supplemented with a complete protease inhibitor cocktail (Roche). After centrifugation, the supernatants were precleared with protein A/G beads at 4 °C for 2 h. Pre-cleared lysates were mixed with a 50% slurry of glutathione-conjugated Sepharose beads (Amersham Biosciences), and the binding reaction was incubated for 4 h at 4 °C. Precipitates were washed extensively with lysis buffer. Proteins bound to glutathione beads were eluted with SDS loading buffer by boiling for 5 min.

For immunoprecipitation, cells were harvested and then lysed in NP-40 buffer supplemented with a complete protease inhibitor cocktail (Roche). After pre-clearing with protein A/G agarose beads for 1 h at 4 °C, whole-cell lysates were used for immunoprecipitation with the indicated antibodies. Generally, 1-4 μg of commercial antibody was added to 1 ml of cell lysates and incubated at 4°C for 8 to 12 h. After the addition of protein A/G agarose beads for 6 h, immunoprecipitates were extensively washed with lysis buffer and eluted with SDS loading buffer by boiling for 5 min.

For immunoblotting, polypeptides were resolved by SDS-polyacrylamide gel electrophoresis (PAGE) and transferred to a PVDF membrane (Bio-Rad). Immuno detection was achieved with specific antibodies. Antibody binding was visualized by chemiluminescence (ECL; Millipore) and detected by a Vilber chemiluminescence analyzer (Fusion SL 3; Vilber Lourmat).

**Quantitative real-time PCR**

Total RNA was extracted from cells using the RNeasy RNA extraction Mini-Kit (Qiagen). cDNA was synthesized using the Enzynomix kit (Enzynomix) and quantitative PCR was performed using gene-specific primer sets (Bioneer) and SYBR Green PCR Master Mix (Roche). Real-time PCR was performed using a QuantStudio™ 3 (ABI), according to the manufacturer’s instructions. Data were normalized against β-actin expression. Relative expression was calculated using the delta-delta CT method. The sequences of the primers used are listed.

| ***Gene*** | ***Sense primer*** | ***Antisense primer*** | ***Size (bp)*** |
| --- | --- | --- | --- |
| mATP5a1 | gccctcggtaatgctattga | gcaatcgatgttttcccagt | 209 |
| mATP5e | tactggcgacaggctggac | ctactccttcttcgagactttcaca | 150 |
| mMFN1 | gctgtcagagcccatctttc | cagcccactgttttccaaat | 195 |
| mMFN2 | gccagcttccttgaagacac | gccagcttccttgaagacac | 208 |
| mOPA1 | gatgacacgctctccagtga | tcggggctaacagtacaacc | 177 |
| mNDUFA9 | actgtgtttggggctacagg | gattgatgaccacgttgctg | 217 |
| mNDUFB8 | ggccgccaagaagtataaca | caccccagttcatcctgagt | 158 |
| mSDHA | acacagacctggtggagacc | ggatgggcttggagtaatca | 156 |
| mSDHB | actggtggaacggagacaag | ttaagccaatgctcgcttct | 248 |
| mUQCRC2 | gtcagagggcttcctgagtg | actcgtcgagaaaaggcgta | 194 |
| mUQCRQ | ggcacgtgatctcctacagc | cgactgctcaaactcctggt | 176 |
| mCOX4L1 | actaccccttgcctgatgtg | gcccacaactgtcttccatt | 188 |
| mCOX6B | ccccaaccagaaccagacta | gatcttcccaggaaatgtgc | 196 |
| mPGC-1α | tcagaaccatgcagcaaacc | ttggtgtgaggagggtcatc | 177 |
| mPGC-1β | tctgccaacggaaacaaagg | gctgctgtcctcaaatacgg | 202 |
| mNRF1 | acagatagtcctgtctgggaaa | tggtacatgctcacagggatct | 99 |
| mNRF2 | gagctagatagtgcccctgg | caggactcacgggaacttct | 169 |
| mTFAM | aagacctcgttcagcatataacatt | ttttccaagcctcatttacaagc | 104 |
| mDRP1 | agaaaactgtctgcccgaga | gctgccctaccagttcactc | 169 |
| mFIS1 | ccggctcaaggaatatgaaa | acagccagtccaatgagtcc | 195 |
| mND1 | ggcccattcgcgttattctt | tcgtaacggaagcgtggata | 197 |
| mPKLR | atctacattgacgacgggct | acattatgctccaccccgaa | 191 |
| mβ-Actin | aagtgtgacgttgacatc | gatccacatctgctggaagg | 222 |

**Cell culture**

Human Colorectal carcinoma Cell Line HCT116 (ATCC-CCL247) and HT-29 cells (ATCC HTB-38), human liver hepatocellular carcinoma Hep G2 (ATCC HB-8065) and Hep3B (ATCC HB-8064), human breast adenocarcinoma cell line MCF7 (ATCC HTB-22) and [MDA-MB-231 (ATCC HTB-26](https://www.atcc.org/Products/All/HTB-26.aspx)) were maintained in DMEM (Invitrogen) containing 10% FBS (Invitrogen), sodium pyruvate, nonessential amino acids, penicillin G (100 IU/ml), and streptomycin (100 μg/ml).

**Cellular fractionation**

Cytosol and mitochondria were isolated from cells using a Mitochondria Fractionation Kit (Active Motif, 40015) or as described previously ^1^. Subcellular fractionated proteins were lysed in buffer containing 2% SDS and boiled with 2x reducing sample buffer for SDS-PAGE.

**Lentiviral shRNA production and transduction**

Lentiviral shRNA production, concentration, titration and transduction were described previously ^2,3^ using the target shRNA plasmid DNA (human PKCα; RHS4531-EG5578, SIRT3; RHS4531-EG23410 and ATP5A1; RHS5086-EG498) were purchased from Open Biosystems. A parallel experiment using a GFP-encoding lentivirus (the pGIPZ lentiviral vector; Open Biosystems) indicated that 80% of cells were successfully transduced by the virus.

**Peptide spot arrays**

The peptide membrane was blocked at RT for 30 minutes in binding buffer containing 5% BSA. Recombinant PKCα (5 nM) was added to 50 mM HEPES, pH 7.4, 100 mM NaCl, 10 mM MgCl_2_, 100 mΜ ATP, 1 mM CaCl2, 6 μCi/ml [γ-^32^P]ATP and incubated at RT for 15 minutes. The membrane was washed three times with 100 mM sodium phosphate pH 7.0, 1 M NaCl, 10 mM EDTA and visualized using phosphorimaging (Fuji phosphor imager). The phosphorylation of each peptide was detected and quantified using Multi Gauge version 3.0 (Fujifilm).

**MTT assay**

Cell viability relative to non-treated group was measured by MTT assay. RAW264.7 and THP-1 cells were seeded for 24 h and culture media was replaced with serum-deficient growth media containing SPG-SH3 or SPG-SC. After incubating for 24 h, 5 mg/ml of MTT (3-(4,5-dimethylthiazol-2-yl)-2,5-diphenyltetrazolium bromide) solution was added in the place of media, and cells were incubated for further 4 h. Then, all the media was removed and the same volume of dimethyl sulfoxide (DMSO) solution was added for 15 min to dissolve the formazan. Using UV/VIS spectrophotometer, each well of the plate was measured at 540 nm to measure relative cell viability

**Measurement of ATP production**

ATP levels were measured by the Luciferin/Luciferase method using the ATP Bioluminescence assay kit (PerkinElmer, 6016943) according to the manufacturer’s instructions. Briefly, ~2 × 10^5^ BMDMs were treated with rGRA8 and washed with PBS. ATP liberation buffer was then added to the cell cultures. Luciferin and Luciferase were mixed together (50 μl, final volume) in a separate vial to which 50 μl of lysate (liberated ATP) from the cell cultures was then added. Luminescence was analyzed after a 30s delay on a FluoroScan luminometer (Labsystems, Helsinki, Finland). An ATP standard curve prepared at the same time according to the manufacturer’s instructions was used to calculate the concentration of ATP in each sample.

**Mitochondrial DNA quantification**

To quantify mtDNA copy number, we measured the mitochondrial (mt) to nuclear (n) DNA ratio, as described previously ^4^. Pyruvate kinase (*Pklr*) was used as a marker for nDNA and NADH dehydrogenase subunit 1 (*mt-Nd1*) for mtDNA. Real-time PCR reactions were performed according to the manufacturer’s instructions (QuantiFast SYBR green PCR master mix; Qiagen, 204052), and thermal cycling was performed in a QuantStudio™ 3 (ABI). The mtDNA content was normalized to the nucleic DNA content. The primer pairs used for PCR are listed in Table.

**Mitochondrial membrane potential measurements**

The mitochondrial membrane potential (ΔΨm) of intact cells was measured as described previously ^4^ with modifications. Cells were washed with PBS and trypsinized. The protein concentration of cells was adjusted to 0.2 mg/ml in DMEM without phenol red (Life Technologies-Invitrogen), FBS, and antibiotics. TMRE (tetramethylrhodamine, ethyl ester; 200 nM, Molecular Probes-Invitrogen, T669) was added to the cell suspension. Cells were incubated at 37 °C for 30 min in the dark. ΔΨm was measured by flow cytometry, and data were analyzed using the FlowJo software. TMRE fluorescence was measured using the FL2 channel (582 nm).

**Enzyme-linked immunosorbent assay**

Mice sera were analyzed for cytokine content using the BD OptEIA ELISA set (BD Pharmingen) for the detection of TNF-α, IL-6, IL-1β, IL-12p40, IL-10, and IL-4. All assays were performed as recommended by the manufacturers.

**Confocal image assay**

GRA8-expressed 293T cells were incubated with 100 nM MitoTracker® mitochondrion-selective probes (Molecular Probes) for 30 min and fixed for 10 min in 4% (vol/vol) paraformaldehyde in PBS and were made permeable for 10 min with 0.1% (vol/vol) Triton X-100 in PBS, then were treated for 1 h at 25 °C with 5% (wt/vol) BSA. Cells were then incubated for 1.5 h at 25 °C with antibody to GST (1:400 dilution). After being washed, cells were further incubated for 1 h at 25 °C with Alexa Fluor 488–conjugated antibody to rabbit immunoglobulin G. For staining of nuclei, the cells were stained with DAPI (4′,6-diamidino-2-phenylindole; Sigma-Aldrich). After mounting, fluorescence images were acquired with a confocal laser-scanning microscope (LSM 780; Zeiss). Fluorescence intensity was measured with ImageJ analysis software (US National Institutes of Health) or Adobe Photoshop CS4 software (Adobe Systems).

**Bacteria count**

Blood and peritoneal lavage fluids were collected from mice by cardiac puncture at indicated time after CLP or bacteria infection. After performing serial dilution of blood, 5 μl of each dilution was plated on blood agar plates. Bacteria were counted after incubation at 37 ºC for 24 h and calculated as counting colony-forming units per whole peritoneal lavage or blood.

**Histology and immunohistochemistry**

For immunohistochemistry of tissue sections, mouse spleens, livers, and lungs were fixed in 10% formalin and embedded in paraffin. Paraffin sections (4 μm) were cut and stained with hematoxylin and eosin (H&E). Histopathologic score was established on the basis of the numbers and distribution of inflammatory cells within the tissues, as well as noninflammatory changes such as evidence of bronchiolar epithelial injury and repair ^5^. The scores were assigned as follows: 0, no inflammation; 1, mild, inflammatory cell infiltrate of the perivascular/peribronchiolar compartment; 2, moderate, inflammatory cell infiltrate of the perivascular/peribronchiolar space with modest extension into the alveolar parenchyma; and 3. severe, inflammatory cell infiltrate of the perivascular/peribronchiolar space with a greater number of inflammatory foci found in the alveolar parenchyma. A board-certified pathologist scored each lung section independently without prior knowledge of the treatment groups.

**REFERENCES**

1. Yang CS, Kim JJ, Kim TS, Lee PY, Kim SY, Lee HM *et al.* Small heterodimer partner interacts with NLRP3 and negatively regulates activation of the NLRP3 inflammasome. *Nat Commun* 2015; **6:** 6115.

2. Koh HJ, Kim YR, Kim JS, Yun JS, Jang K, Yang CS. Toxoplasma gondii GRA7-Targeted ASC and PLD1 Promote Antibacterial Host Defense via PKCalpha. *PLoS Pathog* 2017; **13:** e1006126.

3. Yang CS, Lee JS, Rodgers M, Min CK, Lee JY, Kim HJ *et al.* Autophagy protein Rubicon mediates phagocytic NADPH oxidase activation in response to microbial infection or TLR stimulation. *Cell Host Microbe* 2012; **11:** 264-276.

4. Yang CS, Kim JJ, Lee HM, Jin HS, Lee SH, Park JH *et al.* The AMPK-PPARGC1A pathway is required for antimicrobial host defense through activation of autophagy. *Autophagy* 2014; **10:** 785-802.

5. Kim YR, Hwang J, Koh HJ, Jang K, Lee JD, Choi J *et al.* The targeted delivery of the c-Src peptide complexed with schizophyllan to macrophages inhibits polymicrobial sepsis and ulcerative colitis in mice. *Biomaterials* 2016; **89:** 1-13.
